# PPAR-*γ* Agonists and Their Role in Primary Cicatricial Alopecia

**DOI:** 10.1155/2017/2501248

**Published:** 2017-12-03

**Authors:** Sarawin Harnchoowong, Poonkiat Suchonwanit

**Affiliations:** Division of Dermatology, Faculty of Medicine, Ramathibodi Hospital, Mahidol University, Bangkok, Thailand

## Abstract

Peroxisome proliferator-activated receptor *γ* (PPAR-*γ*) is a ligand-activated nuclear receptor that regulates the transcription of various genes. PPAR-*γ* plays roles in lipid homeostasis, sebocyte maturation, and peroxisome biogenesis and has shown anti-inflammatory effects. PPAR-*γ* is highly expressed in human sebaceous glands. Disruption of PPAR-*γ* is believed to be one of the mechanisms of primary cicatricial alopecia (PCA) pathogenesis, causing pilosebaceous dysfunction leading to follicular inflammation. In this review article, we discuss the pathogenesis of PCA with a focus on PPAR-*γ* involvement in pathogenesis of lichen planopilaris (LPP), the most common lymphocytic form of PCA. We also discuss clinical trials utilizing PPAR-agonists in PCA treatment.

## 1. Introduction

Cicatricial alopecias, or scarring alopecias, are a group of hair loss disorders that are characterized by the permanent destruction of pilosebaceous units. Loss of follicular ostia in the alopecic area and subsequent replacement with fibrous tissue is an important clinical sign [1]. The condition can be classified as primary cicatricial alopecia (PCA) and secondary cicatricial alopecia (SCA). PCA refers to disorders in which the hair follicles are the main targets of destructive inflammatory processes; inflammatory cells destroy the stem cells in the bulge region of hair follicles. In SCA, the hair follicle stem cells are secondarily destroyed by more generalized skin conditions, such as blistering diseases, cancers, trauma, burns, infection, or radiation [1, 2]. PCA is further classified by (predominantly) inflammatory cell type, as shown in Table 1 [3].

**Figure 1:**
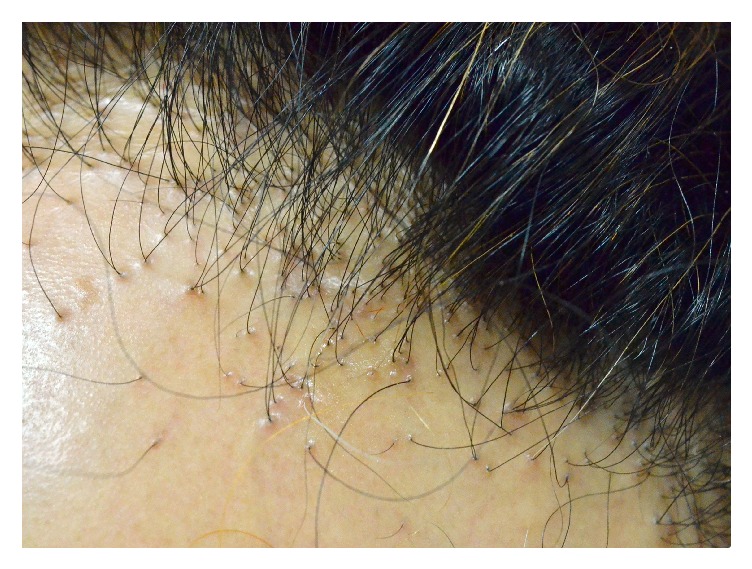
Clinical signs of cicatricial alopecia. The scalp shows a loss of follicular ostia, and the residual hairs show perifollicular erythema and scaling.

**Figure 2:**
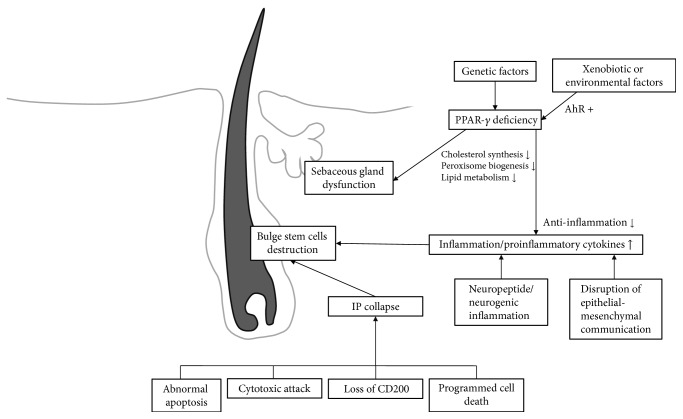
Possible pathogenic pathways in primary cicatricial alopecia.

Like the loss of follicular ostia in the area of alopecia, clinical signs of PCA include evidence of scalp inflammation, for example, perifollicular erythema and perifollicular scales, hair tufting, pustules, skin atrophy, and hypertrophic scarring (Figure 1) [4]. Histopathologically, inflammatory cell infiltration can be observed, distinguished by subtype. Histopathology, together with immunofluorescent staining, can be used to help make a definitive diagnosis of the specific condition [5]. At later stages of the disease, the inflammatory cells will be replaced with fibrous tissues. The etiology and pathogenesis of PCA remain under discussion [6], and there are several hypothesized mechanisms for different types of PCA. This review article summarizes up-to-date knowledge and hypotheses of PCA, especially in pathogenesis and treatment, focusing on one of the latest ideas: peroxisome proliferator-activated receptor *γ* (PPAR-*γ*) involvement in lipid homeostasis within pilosebaceous units. As shown in Figure 2, disruption of this pathway can lead to hair follicle inflammation and permanent destruction [7].

## 2. Pathogenesis of PCA

One of the most widely discussed hypotheses for PCA pathogenesis is hair follicle stem cell destruction. Hair follicles normally regenerate, beginning with the rapidly growing anagen phase, transforming through the catagen phase and resting at the telogen phase [8]. The main requirement for this regeneration capacity is functional epithelial hair follicle stem cells, and these are located within the bulge area at the lower end of the upper half of the hair follicle [9]. However, epithelial stem cells alone cannot initiate the hair cycle; the interaction between hair follicle epithelium and mesenchyme also plays a role. The bulge region is the location of inflammation in scarring alopecia, in contrast to bulb area involvement in other inflammatory nonscarring alopecias, such as alopecia areata [10, 11]. Therefore, the hypothesis that bulge stem cells are associated with hair follicle destruction has merit. Evidence supporting this hypothesis came from a transgenic keratin-15 mouse model in which bulge stem cell destruction led to the permanent loss of hair follicles [12]. Immunostaining of PCA tissues, such as lichen planopilaris (LPP) and chronic cutaneous lupus erythematosus (CCLE), also shows decreased levels of keratin-15, which is almost exclusively limited to the bulge region [13]. Nevertheless, this hypothesis might not adequately explain the pathogenesis of PCA, because dermal papilla-derived and peribulbar dermal sheath cells transplanted into the skin also show the ability to regenerate hair follicles [14].

Immune privilege is another point of interest for investigators. Scientists hypothesize that immune privilege collapse, leading to immunologic responses, could subsequently cause PCA. Immune privilege sites are defined by their low expression levels of major histocompatibility complex (MHC) class Ia and *β*-2 microglobulin and increased levels of immunosuppressive substances such as *α*-melanocyte-stimulating hormone (*α*-MSH), transforming growth factor *β*-1 (TGF *β*-1), and insulin-like growth factor-1 (IGF-1). Normally, the immune privilege area of hair follicles is located around the hair bulb region; this is the area of exclusive autoantigen-induced autoimmunologic attack, as proposed for the pathogenesis of alopecia areata [15]. However, a recent study suggests that the bulge area also demonstrates immune privilege characteristics of reduced MHC-I and II and *β*-2 microglobulin levels and upregulation of cluster of differentiation (CD) 200+, *α*-MSH, TGF-*β*2, macrophage migratory inhibitory factor, and indoleamine-2,3-dioxygenase [16]. This leads to the idea that any immune attack during the failure of this state of immune privilege in the bulge area would lead to epithelial stem cell destruction and, later, permanent loss of hair follicles. Evidence for an initial causal mechanism leading to immune privilege collapse remains inconclusive. One of the upregulated potent immune-regulatory glycoproteins, CD200 is markedly expressed in the bulge area [16, 17]. CD200 shows anti-inflammatory effects and is suspected to be the hair follicle “no danger” signal [18]. Danger/no danger is the latest proposed model of immunologic response; when presented cells are recognized as dangerous invaders, the immune response will be activated [19]. In a CD200-deficient skin model, hair follicles showed inflammation that caused immune-mediated alopecia, correlating to a mouse model showing that CD200 knockdown mice suffer from peri- and intrafollicular inflammation and terminally scarring alopecia [20]. Other substances found to be involved in the maintenance of immune privilege in the hair follicle are somatostatin and programmed death ligand 1 (PD-L1). Somatostatin is upregulated and strongly expressed in the hair follicle outer root sheath relative to the epidermis [21]. When somatostatin is activated, levels of proinflammatory cytokine-like interferon-*γ* (IFN-*γ*) are diminished, leading to the hypothesis that somatostatin has a role in immune privilege preservation. PD-L1 has also been found to have a role in immune privilege maintenance. This substance is highly expressed in dermal papillae and the dermal sheath cup area and epithelium cultured with PD-L1 shows lower levels of IFN-*γ* [22]. Neuroendocrine substances, such as *α*-MSH, prolactin, and thyrotropin-releasing hormone (TRH), are also believed to contribute to the maintenance of hair follicle immune privilege [23].

Besides stem cell destruction, epithelial-mesenchymal inhibition is hypothesized to be one of the mechanisms behind PCA pathogenesis. Epithelial-mesenchymal communication is another essential component for hair follicle cycling, and primary inflammation events cause disruption of this communication. This hypothesis holds that inflammation can attack any region of the hair follicles and is not restricted to the bulge region. However, it could not be confirmed that epithelial-mesenchymal communication failure is the primary event of the disease [24].

In CCLE, cytotoxic cell-mediated hair follicle destruction is hypothesized to be one of the pathogeneses of the disease. Early histologic findings showed that CD4 predominates CD8 in lesional skin [25, 26], and levels of cutaneous lymphocyte antigen (CLA) and cytotoxic marker granzyme B were higher in CCLE scar tissue [26]. These findings suggest that CD4 might invade the follicular epithelium, induce inflammation and apoptosis, and cause scarring at later stages of the disease. IFN-*γ*, or type 1 IFN, is believed to be a possible responsible proinflammatory cytokine. When IFN type 1 is activated, it induces production of various inflammatory chemokines, including CXCL9 and CXCL10, recruits chemokines such as CLA, E-selectin, CCR4, and CXCR3 to diseased skin, and causes local inflammation [27]. Apoptosis is one of the pathogenic processes in CCLE. Fas ligand, an essential component of the apoptotic mechanism, is increasingly expressed in CCLE skin compared to controls, and decreased anti-Bcl-2 staining is evidence of significant apoptosis in CCLE [28]. However, it remains unknown what stimulates the IFN response and apoptosis induction. In a recent study, LPP also showed these characteristics of immune privilege collapse together with cytotoxic cell-mediated follicle destruction. Thus, there is a possibility that LPP might also be an autoimmune hair disorder, similar to alopecia areata, but the location of immune attack is at the bulge region [29].

Another proposed hypothesis for PCA pathogenesis is sebaceous gland dysfunction. In an asebia mouse model with defective stearoyl-CoA desaturase-1 (SCD1), mice exhibit scarring alopecia, sebaceous gland atrophy, and abnormal sebum production [30]. Sebaceous gland atrophy and defective sebum production are alleged to be the causes of foreign body reaction, inflammation, and permanent hair follicle destruction. PPAR-*γ* deficiency has also been raised as a possible pathogenetic mechanism of PCA, as demonstrated by a PPAR mouse model [31]. PPAR-*γ* mediates lipid metabolism and inflammation, especially in pilosebaceous units. Hence, defective PPAR-*γ* could lead to failure of pilosebaceous units and cause permanent hair follicle loss [31]. The following sections of this review will discuss PPAR-*γ* and PCA association in greater depth.

Other possible causes of PCA have been proposed, but no definitive mechanism explaining how these pathogens cause the disease has been found. For example, LPP was found to be associated with exposure to gold [32]. Other drugs that have been associated with PCA are hepatitis B vaccines causing Graham-Little-Piccardi-Lasseur syndrome [33], anticonvulsants and cyclosporine causing acne keloidalis nuchae (AKN) [34–36], and imatinib causing follicular mucinosis [37]. UV exposure is related to erosive pustular dermatosis (EPD) [38], and folliculitis decalvans (FD), AKN, and EPD can be koebnerized by trauma [39]. A series of cases of LPP and FFA following hair transplant or facelift surgeries have been reported, without describing a specific mechanism [40–42].* Staphylococcus aureus *is the main pathogen in FD [43]. Genetic factors also play a role in PCA development, and there are multiple genes associated with PCA [11]. African ancestry is associated with AKN [44]. Stress and neuropeptides also have an impact on alopecia development [45, 46]; however, they will not be discussed here as they are not the primary objective of this review article.

## 3. Molecular Structure and Function of PPARs

**Figure 3:**
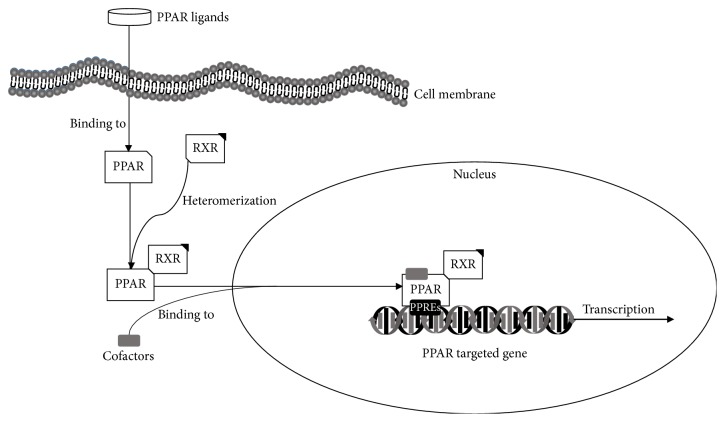
Activation of PPAR-RXR complex, leading to PPAR-targeted genes transcription.

PPARs are members of the ligand-activated nuclear receptors superfamily, regulating the transcription of various genes. PPARs are named for their function in peroxisome proliferator substance activation. There are three isoforms, PPAR-*α*, PPAR-*β*/*δ*, and PPAR-*γ*, each encoded by different genes and distributed differently [47]. PPARs are transferred into the nucleus and heterodimerized with retinoid X receptors (RXR) [48]; then heterodimeric PPAR complexes can bind to specific DNA sequences in the promotor region of target genes containing peroxisome proliferator response elements (PPREs), in the absence of ligands. Several PPAR ligands, both endogenous and exogenous, have been discovered. When specific ligands trigger the PPAR complex, conformational changes occur which lead to transcription of the targeted genes and subsequent translation into specific proteins (Figure 3) [49, 50]. Endogenous ligands for all PPARs include fatty acids and eicosanoid acids. Binding is specific to different types of PPARs; PPAR-*α* and PPAR-*β*/*δ* can bind both saturated and unsaturated fatty acids, but only polyunsaturated fatty acids bind PPAR-*γ* [51]. In addition, exogenous ligands, such as thiazolidinediones (TZDs) and fibrates, have recently been developed for the treatment of various PPAR-associated diseases [7].

**Figure 4:**
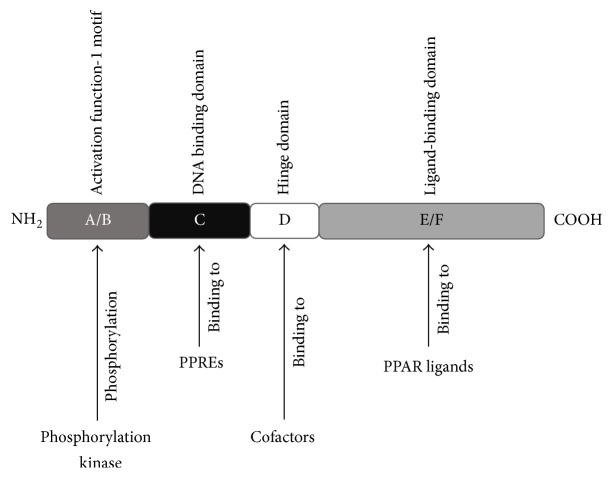
Diagram of the functional domain of PPARs.

PPARs are expressed in several components of human skin (Table 2) [7]. They are composed of 4 main functional domains: the A/B domain that contains the activation function-1 motif, a target of phosphorylation kinase, the C domain, a DNA binding domain that functions as a binding site for PPREs, the D domain, a hinge domain functioning as the docking site for cofactors, and the E/F domain, a ligand-binding domain that functions as a binding site for specific ligands, activating PPARs and promoting target gene expression (Figure 4) [56].

PPAR-*α* is located on chromosome 22q12.2–13.1. Its main function is to regulate fatty acid homeostasis, both mitochondrial and peroxisomal, especially fatty acid catabolism and *β*-oxidation. Apart from fatty acid regulation, it is also believed to have anti-inflammatory properties; data has shown that PPAR-*α* inhibits proinflammatory gene expression in vascular smooth muscles, leading to reduced prevalence of atherosclerosis. PPAR-*α* is significantly expressed in tissues with high fatty acid oxidation, including the liver, heart, and skeletal muscles [57, 58]. Cells and tissues with lower expression include brown adipose tissue, kidneys, adrenal glands, macrophages, smooth muscles, and endothelial cells [59]. PPAR-*α* is proposed as one of the pathogeneses of various hepatic conditions. The important endogenous ligands for PPAR-*α* are unsaturated fatty acids, eicosanoids, leukotriene derivatives, and very low-density lipoprotein (VLDL) [7, 60–63]. Important synthetic PPAR-*α* agonists are known lipid lowering agents, such as the fibrate group [64].

The role of PPAR-*β*/*δ* has not been completely elucidated. There is a lack of information about its function and characteristics due to its ubiquitous expression [57, 58]. It is located on chromosome 6p21.1–21.2 and is expressed widely throughout body, highly so in adipose tissue. It has also been found in the liver, cardiac and skeletal muscles, the brain, kidneys, and colon and in vascular and epidermal tissues [59]. PPAR-*β*/*δ* is involved with metabolic diseases; it increases lipid oxidation in adipose cells, skeletal muscles, and the heart and improves HDL and insulin resistance status. Other functions include cell proliferation/differentiation induction, weight gain limitation, and inflammatory inhibition, especially in the vascular walls [65]. Its endogenous ligands are unsaturated fatty acids, carbaprostacyclin, and VLDL [7].

PPAR-*γ* is the most widely discussed PPAR and is our focus in this review article. PPAR-*γ* is located on chromosome 3p25. It is the most important PPAR; numerous studies relate it with the pathogeneses of different diseases. Its expression is mainly in adipose tissue and sebocytes, but it is also found in the liver, intestinal system, kidneys, retinas, spleen, immune system, skin, sebaceous glands, and thyroid cells and is sparsely expressed in muscles [57–59, 66–70]. PPAR-*γ* helps to maintain glucose metabolism via insulin sensitization and regulate adipocyte differentiation and lipid storage and acts as an anti-inflammatory agent. Essential fatty acids and their derivatives, for example, eicosanoid and prostaglandin J2, are common ligands for PPAR-*γ* [7, 71]. Other recognized PPAR-*γ* agonists are TZDs [49] and nonsteroidal anti-inflammatory drugs (NSAIDs) [72]. Once activated, PPAR-*γ* produces 7 mRNA transcripts that are later transcribed into 3 proteins [73]. PPAR-*γ*1 transcript is found in adipose tissue, liver, pancreatic *β*-cells, intestines, bone, kidney, adrenal glands, vascular cells, and few in skeletal muscles. PPAR-*γ*2 is almost exclusively found in adipose tissues under normal circumstances. PPAR-*γ* 3, 6, and 7 are also found in adipose tissues, and nearly all PPAR mRNAs are found in macrophages [74]. PPAR-*γ*1 protein is translated from transcripts 1, 3, 5, and 7, PPAR-*γ*2 protein from transcripts 1 and 2, and PPAR-*γ*4 protein from transcripts 4 and 6 [73, 75, 76]. PPAR-*γ*2 protein is involved in adipogenesis with synergistic assistance from PPAR-*γ*1 [77].

As PPAR-*γ* is found discretely through multiple systems, defects are thought to be associated with the pathogenesis of various diseases. A prominent association has been found between PPAR-*γ* expression and multiple cardiovascular diseases, including hypertension, atherosclerosis, heart failure, diabetic cardiomyopathy, angiogenesis, and cardiac fibrosis [78, 79]. It also participates in many metabolic diseases, such as diabetes mellitus, obesity, and weight regulation through its effects on lipid homeostasis and insulin sensitivity [80]. Through its anti-inflammatory properties and immune system involvement, it is also believed to have a role in systemic sclerosis, autoimmune thyroid diseases, astrocyte-associated neurodegenerative diseases, and LPP, the type of scarring alopecia that is the focus of this article; countless other associations remain to be discovered in the future [31, 81–83].

## 4. The Role of PPAR-***γ*** in PCA Pathogenesis

**Figure 5:**
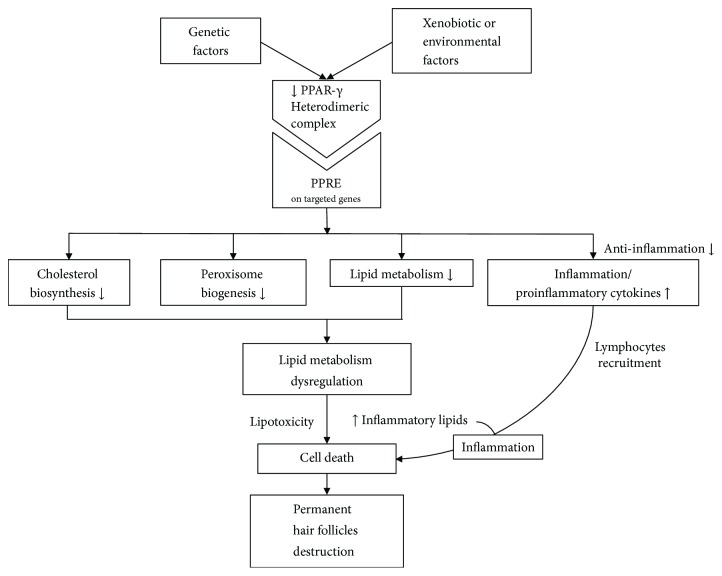
The role of PPAR-*γ* in the pathogenesis of primary cicatricial alopecia.

As mentioned above, PPAR-*γ* is believed to be one of the possible pathogeneses of PCA. PPAR-*γ* is linked to lipid homeostasis and inflammatory regulation of various systems including sebaceous glands. Sebaceous glands function in sebum production and are critical for hair follicle cycling [84, 85]. They are formed together with hair follicles, producing pilosebaceous units which are frequently the target sites of inflammatory reactions that result in sebaceous gland dysfunction (Figure 5) [1]. Several mouse models with dysfunctional sebaceous glands present alopecia phenotypes, mimicking scarring alopecia [11, 30, 31]. However, in humans, sebaceous gland atrophies present differently in each patient with PCA, so it is controversial to call sebaceous gland failure the primary event of the disease [31]. Nevertheless, there is one important factor linking all events together and that is PPAR-*γ*.

In skin biology, PPAR-*γ* is expressed in various structures, including epidermal keratinocytes, dendritic cells, T lymphocytes, and hair follicle outer root sheaths and is almost exclusively expressed in fibroblasts, mast cells, hair follicle inner root sheaths, and active sebocytes [7]. PPAR-*γ* is found abundantly expressed in active or young sebocytes due to its roles in sebocyte and keratinocyte differentiation and epidermal lipid homeostasis [68, 86], while it is sparsely expressed during terminal sebaceous differentiation [7].

PPAR-*γ* is evidently a primary defect of LPP pathogenesis by comparison of histopathology, gene expression, gene activity, and other profiling methods of the scalp of normal subjects and nonlesional and lesional areas of LPP. First of all, clinical samples from the active edge of early diagnosed LPP, unaffected scalp, and normal hosts show that the active area comprises follicular erythema and scaling, and features easily pulled off anagen hairs, but unaffected areas show features similar to that of normal scalp. Histopathology of unaffected areas shows only mild lymphocytic infiltration with minimal atrophic sebaceous glands, compared to dense lymphocytic infiltration and follicular involvement in active phase, and fibrosis and scarring of follicles at the terminal stage [31].

Gene expression comparison among affected and unaffected areas and normal scalp shows that some genes are downregulated in hair follicle cycling, lipid homeostasis, and peroxisome biogenesis, including PEX3 and PEX16, and some genes involved in the inflammatory cascade and apoptotic pathways are upregulated. Expression of genes involved in fatty acid metabolism and desaturation and cholesterol synthesis is downregulated in both lesional and nonlesional areas, so we could hypothesize that these events take place earlier in the course of the disease. In contrast, CD40, SPG21, and reticulum aminopeptidase-1 (ARTS-1) are the only three factors found to be upregulated in nonlesional scalp areas, being part of cytochrome P450 and xenobiotic NF-kB pathways. Both pathways are believed to be an important part of early pathogenesis. Macrophage activation, T-cell lymphocyte chemotaxis, and apoptosis occur later on [31].

Peroxisomes function in various metabolic activities, including lipid metabolism. They require peroxins (PEXs) for their biogenesis, especially PEX3 and PEX16, which are both quite specific to PPAR-*γ*. Evidence from keratinocyte culture of all PPAR isoform agonists with PEX3 and PEX16 shows that only PPAR-*γ* agonists can stimulate PEX3 and PEX16 expression. This correlates with RT-PCR results of affected tissues showing significantly decreased levels of PPAR-*γ* while other PPAR levels remained unchanged. PEX3 expression was downregulated in both lesional and nonlesional areas of LPP scalp; in contrast PEX16 and PEX22 were downregulated only in affected areas. It might be possible to conclude that PEX3, or peroxisome biogenesis, is one of the earliest events of disease progression. Immunofluorescence staining shows that the disappearance of peroxisomes is an early event because sebaceous glands are still found stained in early lesions [31].

Using analysis of Positions and Patterns of Elements of Regulation, PPREs are involved in all downregulated genes. COX2 expression is found to be upregulated. COX2, an inflammation regulation gene, and PPAR-*γ* have negative feedback loop as evidenced by COX2 inhibition after application of PPAR-*γ* agonists to the hair follicle outer root sheath. Supporting these lines of evidence with lipid profile evaluation, levels of cholesterol ester and sapienic acid were decreased. On the contrary, levels of triglycerol and arachidonic acid were found to be increased within lesions [31].

The next point to consider is the trigger factor for PPAR-*γ* dysfunction. A xenobiotic pathway was found to be upregulated in the assay mentioned above. Aryl hydrocarbon receptor (AhR) is the xenobiotic or environmental trigger of PPAR-*γ* suppression. Supporting evidence from microarray data shows increased expression of the CYP1A1 gene, which is associated with AhR. Thus, environmental factors could be a trigger factor of this condition [31].

The last evidence to support the role of PPAR-*γ* in LPP pathogenesis comes from mouse models, including both PPAR-*γ* knock out and Gsdma3^Dfl^/+ mouse models [31, 87]. These presented phenotypes and molecular characteristics consistent with LPP. More recently, another study highlighted the relationship between PPAR-*γ* and hair follicle cycling by showing the effect of PPAR-*γ* modulation on proliferation of hair follicle progenitor cells, keratin-15, and keratin-19 [88].

Despite all the evidence supporting the hypothesis of a role for PPAR-*γ*, several points should be noted. The RNA extraction, gene-profiling, and other microarray sampling in the study mentioned above were performed on whole tissue samples, not from individual hair follicles and sebaceous glands. It could not be definitively concluded that these changes actually occur in our sites of interest, because there could be some interference and overshadowing from other tissues. Further analysis of PPAR-*γ* protein levels, especially in hair follicle and sebaceous glands, would be more specific and helpful to support the hypothesis that PPAR-*γ* disruption is the most important and earliest event of LPP pathogenesis. Other small comments must be made on xenobiotic effects and mouse models. In the study mentioned above, confounding effects were not included in significance analysis, so these could potentially affect the results [89]. The major objection to a role for PPAR-*γ* in LPP pathogenesis comes from a recent study comparing levels of PPAR-*γ* in lesional and nonlesional biopsies. This study specifically assessed PPAR-*γ* in bulge epithelium, the site of epithelial hair follicle stem cells. The results showed no difference in PPAR-*γ* expression in these areas and this raises the question of why globally decreased expression of PPAR-*γ* would affect only focal areas of the hair follicle and not the entire follicle [29]. Thus, we hesitate to conclude that PPAR-*γ* plays a major role, but it might be said that PPAR-*γ* dysfunction precipitates early stage hair follicle changes that lead to inflammatory recruitment or autoimmune attack at bulge stem cells.

## 5. PPAR-***γ*** Implications in PCA

TZDs, PPAR-*γ* agonists, have been discovered to be useful in diabetes mellitus [64]. They increase insulin sensitivity leading to a reduction in blood glucose levels. They are also used as anti-inflammatory agents, inhibiting the secretion of inflammatory-related cytokines such as IL-1, IL-6, IFN-*γ*, CXCL 10 [90], and CXCR 3 [72, 91, 92]. In addition to diabetes mellitus, many TZDs are used to treat ulcerative colitis, rheumatoid arthritis, atherosclerosis, asthma, systemic lupus erythematosus (SLE), renal fibrosis, and psoriasis, and other inflammatory skin diseases [7, 93–98].

Linking PPAR-*γ* to the pathogenesis of LPP, several pieces of information focusing on TZDs, PPAR-*γ* agonists, as alternative treatment options for PCA have been reported. Information from the 2011 cicatricial alopecia symposium revealed that pioglitazone, a TZD, could improve LPP symptoms, both clinically and pathologically, in more than half of patients [99]. In addition, 4 clinical trials reported the efficacy and safety of using pioglitazone in patients with PCA who failed to respond to ordinary treatments. All trials are summarized in Table 3.

The first case report of LPP was published in 2009. A 47-year-old man was diagnosed with LPP and received a series of treatments, including oral prednisolone, oral hydroxychloroquine, oral antibiotic, mycophenolate mofetil, intralesional corticosteroid injection, topical tacrolimus, topical high-potency corticosteroid, and antiseborrheic shampoo. He later received oral pioglitazone at 15 mg/day dose as an alternative regimen. After having treatment for 8 months, he recovered fully and remained symptom-free for 1 year after drug discontinuation [52]. In another clinical trial of 24 patients with LPP, half of the group showed improvement after using oral pioglitazone, while 5 achieved disease remission. Four patients dropped out of study due to adverse reaction and intolerability [53]. In 2013, 22 patients with resistant LPP were given oral pioglitazone at a starting dose of 15 mg/day. Only 3 patients showed a good recovery, while the others were considered to show negative effectiveness. Four patients showed clinical improvement but the symptoms relapsed after pioglitazone discontinuation. Two of these four patients were rechallenged but found to be resistant to pioglitazone [54]. In the latest study in 2015, Mesinkovska et al. retrospectively reported a case series of all-female patients with LPP. Patients receiving oral pioglitazone for at least 1 month and follow-up of up to 3 months were included to this study. The initial dose of pioglitazone started at 15 mg/day and stabilized disease progression in nearly 73% of patients, while 6 patients out of 22 showed hair regrowth. Disease relapsed in two patients (9%) after drug discontinuation. The most common side effect in this study was edema of the lower extremities (50%) leading to 9 out of 22 patients withdrawing from the study [55].

The results of these trials show the same trend that pioglitazone at least helps to stabilize the disease. There are conflicting results between trials in terms of improvement, ranging from perceived improvement to great improvement or even resolution. However, the findings from these clinical trials suggest the use of PPAR-*γ* agonists as a treatment option for PCA, especially LPP. TZDs inhibit the inflammatory stages of disease by increasing activation of PPAR-*γ* resulting in inhibition of interleukins, proinflammatory nuclear transcription factors, and proteolytic enzymes. Further prospective, randomized, double-blind, controlled trials with large numbers of subjects will be necessary to confirm the role of PPAR-*γ* in PCA pathogenesis and the efficacy and safety of TZDs in PCA treatment.

## 6. Conclusion

PCA is a diverse group of inflammatory hair diseases involving the destruction of pilosebaceous units and replacement with fibrosis. There are several hypotheses for PCA pathogenesis, including hair follicle stem cell destruction, immune privilege collapse, autoimmune attack, and sebaceous gland dysfunction, among others. It is difficult to prove definitively which event comes first in disease progression and pathogenesis. PPAR-*γ*, part of the nuclear receptors superfamily, plays a remarkable role in PCA pathogenesis. PPAR-*γ* is involved in sebocyte differentiation, lipid homeostasis, peroxisome biogenesis, and inflammatory regulation. It is believed that PPAR-*γ* deficiency, triggered by unknown factors, leads to pilosebaceous dysfunction and failure of peroxisome biogenesis, decreased sebum secretion, and increases in proinflammatory lipid levels. Inflammation occurs and leads to apoptosis of stems cells and hair follicles. However, recent evidence opposing this hypothesis shows no difference in PPAR-*γ* expression between lesional and nonlesional scalp areas of patients with LPP. We might only conclude that PPAR-*γ* disruption has a predisposing role in PCA pathogenesis but is not the key factor. From clinical trials, pioglitazone, a PPAR-*γ* agonist, is effective in stabilizing patients' clinical symptoms. Hence, PPAR-*γ* agonists might be a good alternative choice of treatment in LPP, the lymphocytic PCA. Development of PPAR-agonists is important to increase specificity and improve efficacy in the near future.

## Figures and Tables

**Table 1:** Classification of primary cicatricial alopecia.

Classification of cicatricial alopecia
Lymphocytic
(i) Discoid lesions of lupus erythematosus
(ii) Lichen planopilaris
(a) Classic LPP
(b) Frontal fibrosing alopecia
(c) Graham Little syndrome
(iii) Pseudopelade of Brocq
(iv) Central centrifugal cicatricial alopecia
(v) Alopecia mucinosa
(vi) Keratosis follicularis spinulosa decalvans
Neutrophilic
(i) Folliculitis decalvans
(ii) Dissecting cellulitis
Mixed cell
(i) Acne keloidalis
(ii) Acne necrotica
(iii) Erosive pustular dermatosis

**Table 2:** Peroxisome proliferator-activated receptors (PPARs) in human skin.

Skin components	Type of PPAR expression
Epidermis and dermis	
(i) Epidermal keratinocytes	PPAR-*α*, PPAR-*β*/*δ*, and PPAR-*γ*
(ii) Melanocytes	PPAR-*α*, PPAR-*β*/*δ*, and PPAR-*γ*
(iii) Fibroblasts	PPAR-*γ*
(iv) T lymphocytes	PPAR-*α*, PPAR-*β*/*δ*, and PPAR-*γ*
(v) Langerhan cells	PPAR-*α*, PPAR-*β*/*δ*, and PPAR-*γ*
(vi) Mast cells	PPAR-*β*/*δ* and PPAR-*γ*
Follicular units	
(i) Hair matrix keratinocytes	PPAR-*α*, PPAR-*β*/*δ*, and PPAR-*γ*
(ii) Hair shaft cortex	PPAR-*α*, PPAR-*β*/*δ*, and PPAR-*γ*
(iii) Hair cuticle	PPAR-*α*, PPAR-*β*/*δ*, and PPAR-*γ*
(iv) Inner root sheath	PPAR-*β*/*δ* and PPAR-*γ*
(v) Outer root sheath	PPAR-*α*, PPAR-*β*/*δ*, and PPAR-*γ*
(vi) Dermal papilla cells	PPAR-*α*, PPAR-*β*/*δ*, and PPAR-*γ*
(vii) Connective tissue sheath	PPAR-*α*, PPAR-*β*/*δ*, and PPAR-*γ*
(viii) Sebocytes	PPAR-*α*, PPAR-*β*/*δ*, and PPAR-*γ*

**Table 3:** Clinical trials of pioglitazone in the treatment of primary cicatricial alopecia.

Authors, year	Study type	Treatment	Outcome
Mirmirani and Karnik, 2009 [52]	Case report: (i) 1 patient with LPP	(i) Oral pioglitazone hydrochloride 15 mg/day for 14 months	(i) 2 months: clinical improvement (ii) 6 months: marked decrease of inflammation (iii) 1 year: remained symptom-free

Baibergenova and Walsh, 2012 [53]	Case series: (i) 21 patients with LPP (ii) 2 patients with FAPD (iii) 1 patient with FFA	(i) Oral pioglitazone hydrochloride 15 mg/day, increased to 30 mg/day if there is no ADR (ii) Concurrent treatments were variably used as needed	(i) 5 patients: remission (ii) 12 patients: improvement (iii) 3 patients: no improvement (iv) ADR in 4 patients leading to withdrawal: calf pain, lightheadedness, dizziness and hives

Spring et al., 2013 [54]	Case series: (i) 22 patients with LPP	(i) Oral pioglitazone hydrochloride 15 mg/day for 1 year (ii) Adjuvant treatments were variably used as needed	(i) 3 patients: remission and no relapse (ii) 5 patients: improvement with lower disease activity (iii) 4 patients: improvement but symptoms relapsed (iv) 10 patients: negative result

Mesinkovska et al., 2015 [55]	Case series: (i) 18 patients with LPP (ii) 4 patients with FFA	(i) Oral pioglitazone hydrochloride 15 mg/day for median of 10.5 months	(i) 16 patients: marked improvement (ii) 5 patients: stable of disease (iii) 1 patient: progression of disease (iv) ADR: lower extremities edema, weight gain, dizziness, resistant hypertension, mild transaminitis

LPP: lichen planopilaris, FFA: frontal fibrosing alopecia, FAPD: fibrosing alopecia in pattern distribution, and ADR: adverse drug reactions.
